# Serum Glycoproteome Profiles for Distinguishing Intestinal Fibrosis from Inflammation in Crohn's Disease

**DOI:** 10.1371/journal.pone.0170506

**Published:** 2017-01-23

**Authors:** Ryan W. Stidham, Jing Wu, Jiaqi Shi, David M. Lubman, Peter D. R. Higgins

**Affiliations:** 1 Department of Internal Medicine, Division of Gastroenterology and Hepatology, University of Michigan, Ann Arbor, MI, United States of America; 2 Department of Surgery, University of Michigan Health System, Ann Arbor, MI, United States of America; 3 Department of Pathology, University of Michigan Health System, Ann Arbor, MI, United States of America; University Hospital Llandough, UNITED KINGDOM

## Abstract

**Background:**

Reliable identification and quantitation of intestinal fibrosis in the setting of co-existing inflammation due to Crohn’s disease (CD) is difficult. We aimed to identify serum biomarkers which distinguish inflammatory from fibrostenotic phenotypes of CD using serum glycoproteome profiles.

**Methods:**

Subjects with fibrostenotic and inflammation-predominant CD phenotypes (n = 20 per group) underwent comparison by quantitative serum glycoproteome profiles as part of a single tertiary care center cohort study. Following lectin elution, glycoproteins underwent liquid chromatography followed by tandem mass spectrometry. Identified candidate biomarkers of fibrosis were also measured by serum ELISA, a widely available technique.

**Results:**

Five (5) glycoproteins demonstrated a ≥20% relative abundance change in ≥80% of subjects, including cartilage oligomeric matrix protein (COMP) and hepatocyte growth factor activator (HGFA). COMP (431.7±112.7 vs. 348.7±90.5 ng/mL, *p* = 0.012) and HGFA (152.7±66.5 vs. 107.1±38.7 ng/mL, *p* = 0.031) serum levels were elevated in the fibrostenotic vs. inflammatory CD groups using ELISA. Within the fibrostenotic group, intra-individual changes of candidate biomarkers revealed HGFA levels significantly declined following the resection of all diseased intestine (152.7±66.5 vs. 107.1±38.7 ng/mL, *p* = 0.015); COMP levels were unchanged. Immunohistochemical staining confirmed the presence of COMP in the submucosa and muscularis of resected fibrostenotic tissue.

**Conclusions:**

In this biomarker discovery study, several serum glycoproteins, specifically COMP and HGFA, differ between between predominately inflammatory and fibrostenotic CD phenotypes. The development of blood-based biomarkers of fibrosis would provide an important complement to existing prognostic tools in IBD, aiding decisions on therapeutic intensity and mechanism selection, surgery, and the monitoring of future anti-fibrotic therapies for CD.

## Introduction

Crohn’s disease (CD), an idiopathic inflammatory bowel disease, has a range of phenotypes resulting from the variation in the amount of accumulated bowel damage. Repetitive cycles of intestinal inflammation followed by wound healing cause an accumulation of fibrosis, which itself is an amalgam of excessive extracellular matrix and smooth muscle hypertrophy [1]. Inflammatory and fibrotic changes together contribute to progressive bowel wall thickening, stricture development, and subsequent obstructing and penetrating complications. Fibrostenotic CD complications are major mediators of disease morbidity with 60% of patients requiring surgery within 10 years of diagnosis. While therapeutic advances have dramatically improved the ability to control inflammatory changes, intestinal fibrosis is irreversible and unresponsive to existing medications [2].

Modern therapeutic strategies aim to prevent or halt fibrosis progression prior to the development of obstructive stricturing disease. Escalation of therapeutic intensity and early use of biologics are associated with improved objective inflammatory disease control and reduction (or delay) of surgery and hospitalization [3,4]. However, personalizing the balance of sufficient medical therapy to avoid morbid complications while minimizing medication-related risks is challenging. Common management questions rely on estimations of the present and future contribution of intestinal fibrosis to overall CD-related bowel damage: Will therapeutic intensification durably avoid surgery? What therapeutic intensity at diagnosis is necessary to avoid fibrostenotic complications? Is the existing therapeutic regimen sufficient to avoid fibrostenotic complications? These points highlight the need for methods to objectively measure the presence and ongoing accumulation of intestinal fibrosis and to quantify the relative contribution of inflammatory and fibrotic disease.

Contemporary disease activity assessments capture Crohn’s-related inflammation and instruments are in development for scoring total bowel damage [5]. However, measuring the relative contribution of fibrosis remains difficult. Ileocolonoscopy is unable to interrogate the deep bowel wall, highlighting the need for complimentary tests evaluating the bowel wall with imaging using ultrasound, computed tomography enterography (CTE) and magnetic resonance enterography (MRE). While image-based assessments of disease activity are proving to be strongly correlated with endoscopic activity scores, their ability to confidently discriminate inflammation from fibrosis remains unclear [6]. Further, the prior concepts of high and low bowel contrast enhancement being characteristic of predominantly inflammatory vs. fibrostenotic intestinal disease have been questioned by studies comparing pre-operative imaging with histology [7,8]. Several regulatory and structural proteins involved in fibrosis, including transforming growth factor-β (TGF-β), procollagen, laminin, and fibronectin, have yet to be shown to be practical clinical measures of fibrosis [9].

To address the need for biomarkers of intestinal fibrosis, we compared serum glycoprotein profiles of patients with well characterized fibrostenotic CD refractory to anti-TNF therapy (insufficient improvement to avoid surgery) to those with an inflammatory phenotype. Nearly 50% of serum proteins are known to be glycosylated and are frequently produced and secreted by gastrointestinal tissues [10]. Glycoproteome profiling has successfully identified biomarkers of fibrosis in chronic pancreatitis, nephrosclerosis, and cirrhosis [11–13]. Further, restricting evaluation to the glycoproteome offers benefits of reduced sample complexity, a significant advantage over traditional proteomics. The aim of this study was to identify candidate biomarkers of intestinal fibrosis, and determine if these change after resection of fibrotic segments of intestine in Crohn’s disease.

## Materials and Methods

### Subject selection criteria

Two separate CD phenotypes were recruited, a fibrostenotic cohort and non-stenotic inflammatory cohort, between 2012–2014 at a single academic referral center. All subjects were 18 years of age or older and were required to have a known disease duration of at least 1 year with terminal ileal involvement. CD diagnosis was verified by at least one inpatient and one outpatient ICD-9 code of 555.X and review of medical records by an experienced IBD specialist [14]. In the absence of a gold standard for fibrosis and considering the coexistence of inflammation and fibrosis, best efforts were made to define these phenotypes. The fibrostenotic phenotype cohort was defined as (1) having recent (prior 3 months) imaging evidence of intestinal stricturing by CTE or MRE with bowel wall thickening ≥3mm; (2) imaging evidence of small bowel dilation of ≥3.5cm upstream of stricturing disease, (3) symptoms refractory to anti-TNF therapy, and (4) plans to undergo elective surgical resection of all diseased tissue with a primary anastomosis. The inflammatory phenotype cohort was defined as (1) having a CRP ≥1.0mg/dL, (2) recent CTE or MRE demonstrating active disease ≥5cm in length by mural/mucosal hyperenhancement, but without evidence of stricturing disease or upstream small bowel dilation (≥3.5cm), (3) no history of prior endoscopic dilation. Subjects in the inflammatory phenotype cohort were required to be exposed to be using either anti-TNF therapy or an immunomodulatory. Exclusion criteria for both groups included prior intestinal surgery, a history of ano-rectal stenosis, active perianal fistulizing disease, intra-abdominal abscess, malignancy, or active infectious enteritis/colitis. Additional exclusion criteria included patients with a history of extra-intestinal fibrotic disease, specifically cirrhosis, nephrosclerosis, scleroderma, idiopathic pulmonary fibrosis, or congestive heart failure.

### Clinical data and specimen collection

Age, gender, smoking status, medication history, disease history, and past medical and surgical history were collected at the time of enrollment. Clinical disease activity was measured using the Harvey Bradshaw Index (HBI). Subjects in the fibrostenotic cohort underwent repeated serum collection at three time points, 0–2 weeks prior to surgery (pre-operative), 4–8 weeks (early post-operative) and 16–24 weeks (late post-operative) following surgery. Those in the inflammatory cohort underwent a serum collection at the time of enrollment. A complete blood count (CBC), albumin, and C-reactive protein (CRP), were collected during the enrollment blood draw. Full-thickness resection sections of fibrostenotic bowel underwent standard tissue processing in preparation for immunofluorescent staining.

### Serum glycoproteomics

Serum samples underwent depletion of the top 14 high-abundance proteins using IgY-14 LC5 columns (Sigma, St. Louis, MO). Post-depletion serum proteins and internal standards were labeled with tandem mass tags (TMT) reagents to improve relative protein quantitation, as previously described [15]. Labeled samples were incubated in a lectin-affinity column (using *Aleuria aurantia* lectin, AAL) to bind and then elute fucosylated glycoproteins. TMT-labeled glycoprotein separation was performed by loading samples into an ultra-high performance liquid chromatography system, coupled to a Orbitrap Fusion Tribrid Mass Spectrometer (Thermo Fisher Scientific). Peptides underwent a full mass spectrometer scan (m/z 350.0–1500.0), followed by ten MS/MS scans of the most intense ions from the full spectrum; measurements were performed in triplicate. The acquired MS/MS spectra were searched for in the UniProt human database using SEQUEST in Proteome Discoverer 1.4 (Thermo) to identify individual peptides. Identified peptides were then filtered using a cutoff of a 1% peptide-level false discovery rate (FDR), quantification was performed using reporter ions. See S1 Appendix for expanded laboratory and bioinformatic methods.

### Protein identification by enzyme-linked immunosorbent assay (ELISA)

Proteins of interest for distinguishing inflammatory and fibrostenotic phenotypes were measured by ELISA assays. Proteins assessed included cartilage oligomeric matrix protein (COMP), cholinesterase (CHLE), procollagen C-endopeptidase (POCE), hepatocyte growth factor activator (HGFA), and tenascin-X (TNXB). The ELISA kit for COMP was purchased from BioVendor (Candler, NC, USA). ELISA kits for CHLE and TNXB were purchased from LSBio (Seattle, WA, USA). ELISA kits for POCE and HGFA were purchased from Cloud-Clone Corp (Houston, TX, USA). ELISA assays were performed following the manufacturer’s instructions. The absorbance values were read on a microplate reader (BioTek, Synergy HT) at a wavelength of 450 nm.

### Immunohistochemistry

Gastrointestinal-specialist pathologists reviewed surgical specimens from the fibrostenotic group, and selected sections most representative of fibrostenotic injury. Normal small intestine and colonic sections for comparison were selected by pathologists, sourced from healthy margins of resected diverticular or malignant disease. The purpose of IHC was to determine whether candidate markers were detected within diseased bowel. Formalin-fixed paraffin-embedded (FFPE) tissue sections were dewaxed in xylene and rehydrated through a series of alcohol solutions (100% ethanol twice, 95% ethanol, 75% ethanol, 5 min each) to water. Antigen retrieval was achieved by boiling the sections in a citrate buffer at pH 6.0 (Invitrogen, Grand Island, NY) for 15 min. The tissue sections were then treated with 2% BSA in PBST for 1 hr to block non-specific binding followed by incubation with rat anti-human COMP and HGFA monoclonal antibodies (1:100 dilution, Abcam, Cambridge, MA) overnight at 4°C. The slides were then stained with Alexa Fluor 594-conjugated secondary goat anti-rat IgG (Abcam, Cambridge, MA) at a dilution of 1:150 for 1-hr at room temperature. Nuclei were visualized by DAPI counterstaining.

### Statistical analysis

All analyses were conducted using SAS 9.4 (Cary, NC). Demographic, clinical, and laboratory features were compared between inflammatory and fibrostenotic groups using the Student’s t-test and a Chi-squared or Fisher’s exact test. Protein abundance, relative to internal standards, was compared between the inflammatory and fibrotic state using a two-sample t-test. Supervised reduction of data dimensionality was performed, requiring proteins to be detected in 80% of samples and demonstrate a 20% relative change between phenotypes for inclusion into the analysis. False discovery rates (FDR) were calculated for proteins remaining in the analysis to account for multiplicity of testing, a commonly used alternative to the Bonferroni correction in proteomic and microarray studies [16]. For the LC-MS/MS discovery work, we selected proteins with an FDR ≤0.10 to undergo ELISA verification. For analysis of within-individual change of protein abundance following surgical resection of fibrostenotic tissue, the paired t-test was used with FDR correction. The sensitivity, specificity, and area under the receiver operating characteristic (AUROC) curve were reported for proteins able to be detected by ELISA using logistic regression.

### Ethical considerations

The study protocol, risks, and benefits were discussed with all Subjects, who provided written consent to participate in this study. The study protocol and informed consent process were approved by the University of Michigan Institutional Review Board.

## Results

### Patient cohort characteristics

A total of 28 subjects were recruited into the fibrostenotic cohort, with 7 patients excluded for failing to provide all three blood samples; 1 patient was excluded after identifying small intestinal adenocarcinoma within the surgical specimen. Within the inflammatory phenotype cohort, 20 subjects meeting selection criteria were enrolled. Comparing demographic and clinical features between groups, the fibrostenotic group was significantly older (46.9 vs. 33.3 yrs., *p =* 0.006) and had a longer mean disease duration (18.2 vs 9.0 yrs., *p* = 0.004). Both cohorts were overwhelmingly Caucasian (85% vs. 90%, *p* = 0.632). Clinical activity scores as assessed by the Harvey-Bradshaw Index (HBI), perianal disease and smoking history, current and historical medication use, and laboratory parameters did not significantly differ between groups (Table 1). All subjects demonstrated evidence of active inflammation based on a C-reactive protein (CRP) level ≥0.5 mg/dL. While the fibrostenotic group exhibited a trend of greater inflammatory activity by mean CRP (2.3 vs. 1.9 mg/dL, *p* = 0.150), the difference was not statistically significant. The active use of combination immunosuppression was similar between fibrostenotic and inflammatory phenotype subjects (25% vs. 20% *p* = 0.705). While all fibrostenotic phenotype subjects were required to have had inadequate response to anti-TNF therapy to avoid surgery, 75% remained on anti-TNF therapy at 8 weeks prior to surgery. Reasons for continuing anti-TNF included preventing progression of disease and continued symptomatic benefits, despite the therapeutic improvements afforded being insufficient to prevent the need for surgery. The median duration of anti-TNF use did not differ between the fibrostenotic (31.4 months, range 14.2–164.3) and inflammatory phenotypes (38.6 months, range 4.7–122.5, *p* = 0.837). Among those with fibrostenotic disease, the mean length of electively resected intestine was 32.6±22.9 cm. All subjects had severe fibrostenotic disease within the resected specimen based on the original histologic report.

**Table 1:** Patient characteristics.

		Fibrostenotic *n* = 20	Inflammatory *n* = 20	*p*-value
		n (%), Mean (SD)	n (%), Mean (SD)	
**Gender, male**		10 (50.0%)	10 (50.0%)	0.999
**Age, yrs**		46.9 (±12.94)	33.3 (±12.34)	**0.006**
**Disease Duration, yrs**		18.2 (±11.98)	9 (±5.57)	**0.004**
**History of Perianal Fistula**		4 (20.0%)	2 (10.0%)	0.661
**History of Smoking**		7 (35.0%)	11 (55.0%)	0.341
**HBI Score**		9.95 (±5.69)	7.75 (±4.12)	0.174
**Medication Use**				
**Anti-TNF**	*Active*	15 (75.0%)	13 (65.0%)	0.731
	*History*	20 (100.0%)	15 (75.0%)	0.408
**Thiopurine**	*Active*	11 (55.0%)	7 (35.0%)	0.341
	*History*	17 (85.0%)	18 (90.0%)	0.633
**Methotrexate**	*Active*	1 (5.0%)	2 (10.0%)	0.548
	*History*	3 (15.0%)	4 (20.0%)	0.677
**Prednisone**	*Active*	6 (30.0%)	3 (15.0%)	0.451
**Laboratory Data**				
**Hemoglobin**		12.7 (±2.24)	13.7 (±1.16)	0.118
**Albumin**		4.1 (±0.52)	4.2 (±0.35)	0.449
**White Blood Cells**		8 (±3.21)	7.5 (±1.57)	0.576
**Platelets**		304.6 (±110.6)	299.7 (±91.3)	0.884
**C-Reactive Protein**		2.3 (±3.31)	1.9 (±0.84)	0.15

### Glycoproteins differentiating predominately fibrostenotic and inflammatory phenotypes

Of a total of 1063 proteins identified, 194 proteins were detected in ≥80% of fibrostenotic group subjects. Of the frequently detected proteins, 24 glycoproteins exhibited a relative abundance change of ≥20% between inflammatory and fibrostenotic groups, composing the set of proteins for analysis. Of those glycoproteins demonstrating ≥20% change in relative abundance between fibrotic and inflammatory groups, 5 exhibited an FDR of ≤0.10, and included cartilage oligomeric matrix protein (COMP) and hepatocyte growth factor activator (HGFA, Table 2). The candidate biomarkers identified were detected by LC-MS/MS in all serum samples. Within the fibrostenotic group, correlations between targeted biomarkers and the length of resected specimen were poor and non-significant. Among all subjects (both inflammatory and fibrostenotic) there was an unsurprising correlation between protein abundance by mass spectrometry and the duration of disease for most targeted biomarkers (except tenascin-X), though the correlations were weak (COMP *r* = 0.1996, *p* = 0.0233; procollagen c-endopeptidase *r* = 0.1888, *p* = 0.0300; cholinesterase *r* = 0.1774, *p* = 0.0321, HGFA *r* = 0.1655, *p* = 0.0391). Age, gender, medication use, and HBI score were not correlated with the abundance of any of the targeted biomarkers. A weak but significant positive correlation between albumin levels and both cholinesterase (*r* = 0.2437, *p* = 0.0104) and HGFA (*r* = 0.1608, *p* = 0.0423) was observed; otherwise, there was no correlation between CRP, WBC, PLT, and HGB among the biomarkers investigated.

**Table 2:** Glycoproteins differentiating inflammatory and fibrostenotic phenotypes.

Candidate Biomarker	Fibrostenotic Expression Ratio (SD)	Inflammatory Expression Ratio (SD)	Relative Change	FDR
Cartilage oligomeric matrix protein	0.877 (0.307)	0.649 (0.240)	1.350	0.0457
Procollagen C-endopeptidase	0.919 (0.401)	0.663 (0.168)	1.386	0.0580
Cholinesterase	0.918 (0.288)	0.767 (0.184)	1.198	0.0750
Tenascin-X	1.029 (0.399)	0.809 (0.175)	1.272	0.0820
Hepatocyte growth factor activator	0.910 (0.345)	0.754 (0.190)	1.207	0.1055

Of 24 glycoproteins found in ≥80% of samples with a relative abundance change between inflammatory and fibrostenotic phenotypes of ≥20%, the candidate biomarkers identified are shown. Mean expression is reported as a ratio relative to constitutive proteins found in each sample, with relative change between fibrostenotic and inflammatory groups shown; adjusted p-values (false discovery rate, FDR) are provided.

### Glycoproteins demonstrating change following resection of fibrostenotic tissue

Glycoproteins demonstrating a within-individual change in relative abundance between pre-operative (high-fibrotic burden) and late post-operative states (following removal of fibrostenotic intestine, 16–24 weeks post-operative) are shown in Table 3. Comparison of pre-operative and early post-operative serum (4–8 weeks post-operative) did not reveal significant changes in the identified proteins, though this is within a period of ongoing surgical wound healing. Functional and pathway ontology analysis demonstrated that these proteins are involved in protease activity (aminopeptidase, a metalloprotease), extracellular matrix production (lumican), and cellular adhesion (cadherin-5). Of the candidate biomarkers that also differentiated inflammatory from fibrostenotic phenotypes, COMP and HGFA both demonstrated within-individual relative abundance reduction following surgical resection (0.8502±0.240 and 0.855±0.181, respectively), though the difference was not statistically significant following adjustment for multiplicity of testing.

**Table 3:** Glycoproteins demonstrating change following resection of fibrostenotic disease.

Candidate Biomarker	Pre-Op vs. Early Post-Op Relative Change (SD)	Early Post-Op vs. Late Post-Op Relative Change	Pre-Op vs. Late Post-Op Relative Change	FDR
Aminopeptidase	0.883 (0.199)	0.976 (0.119)	0.836 (0.191)	0.0266
Polymeric immunoglobulin receptor	0.830 (0.157)	0.930 (0.222)	0.750 (0.214)	0.0381
Cartilage oligomeric matrix protein	0.978 (0.238)	0.945 (0.230)	0.850 (0.240)	0.0514
Hepatocyte growth factor activator	1.089 (0.172)	0.931 (0.152)	0.859 (0.181)	0.0555
Cadherin-5	0.985 (0.221)	0.908 (0.187)	0.894 (0.240)	0.0892
Lumican	1.003 (0.318)	0.992 (0.274)	0.863 (0.274)	0.0948
Peptidase inhibitor 16	1.002 (0.229)	0.992 (0.166)	0.866 (0.165)	0.1138

Of the glycoproteins present in at least 80% of serum samples, the 7 candidate markers demonstrating a within-individual relative change following surgical resection of fibrostenotic disease are shown. Paired pre-operative (week -2-0), early post-operative (week 4–6) and late post-operative (week 12–24) glycoprotein profiles were compared; adjusted *p*-values (by false discovery rate, FDR) are provided. While unclear whether presented markers are reflective of fibrosis or instead post-operative reduction of overall fibro-inflammatory burden, COMP and HGFA trends of elevated levels being associated with fibrotic phenotypes suggest these may be reflective of disease dominated by fibrostenotic changes.

### Protein quantification of candidate biomarkers differentiating inflammation and fibrostenotic disease by ELISA

**Fig 1:**
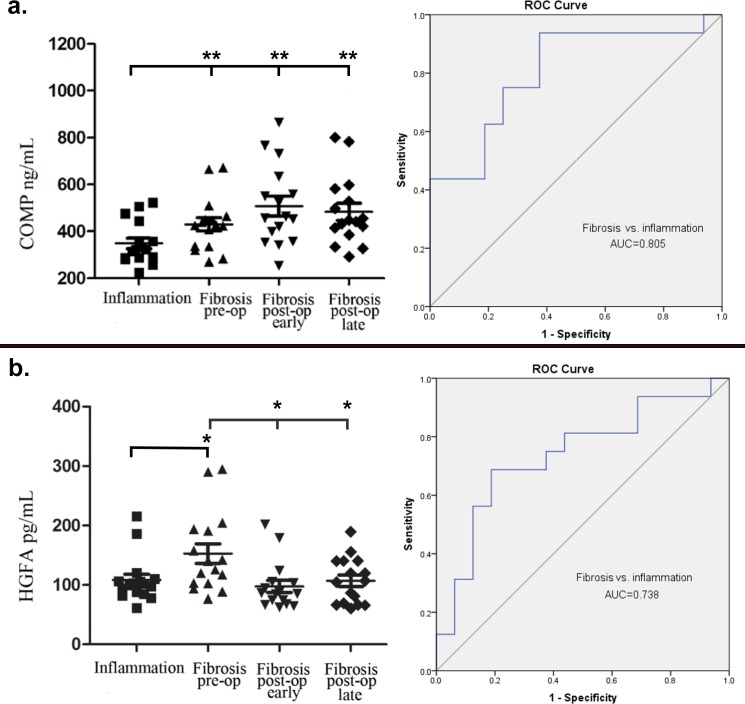
ELISA quantitation of candidate proteins differentiates inflammatory and fibrostenotic disease. ELISA detected significant differences in the concentration of COMP (a) and HGFA (b) between inflammatory and fibrotic states (*p < .01, ** *p* < .001). The AUROC for discriminating inflammatory from fibrostenotic disease was 0.805 using COMP and 0.738 using HGFA. While within-individual paired comparison did not reflect a change in COMP levels following resection of fibrotic tissue, pre-operative elevated HGFA declined following resection.

COMP, procollagen C-endopeptidase (POCE), cholinesterase, tenascin-X and HGFA underwent further assessment and quantification by ELISA. While a significant difference in the relative abundance of POCE, tenascin-X, and cholinesterase between inflamed and fibrostenotic groups was detected by mass spectrometry, no difference between phenotypes was detected by ELISA. COMP quantitation by ELISA was significantly greater in predominantly fibrostenotic compared to inflammatory subjects (431.7±112.7 vs. 348.7±90.5 ng/mL, *p* = 0.012). COMP levels by ELISA remained elevated in the late post-operative period compared to the inflammatory group (483.9±146.9 vs. 348.7±90.5 ng/mL, *p* = 0.004, Fig 1A). However, there was significant COMP decline within fibrostenotic subjects following bowel resection (*p* = 0.186). The AUROC of the logistic regression model for discriminating inflammatory from fibrostenotic phenotypes was 0.805 using COMP protein quantitation by ELISA. HGFA, which had marginally significant abundance differences between phenotypes by mass spectrometry, was shown to be elevated in the fibrostenotic vs. inflammatory phenotypes by ELISA (152.7±66.5 vs. 107.1±38.7 pg/mL, *p* = 0.031, Fig 1B). Further, comparing within-individual changes between pre-operative and late post-operative serum, HGFA significantly declined following resection of fibrostenotic disease (152.7±66.5 vs. 107.1±38.7 pg/mL, *p* = 0.015). The AUROC of the logistic regression model for discriminating inflammatory from fibrostenotic phenotypes was 0.738 using HGFA protein quantitation by ELISA.

### Histology and staining comparison

**Fig 2:**
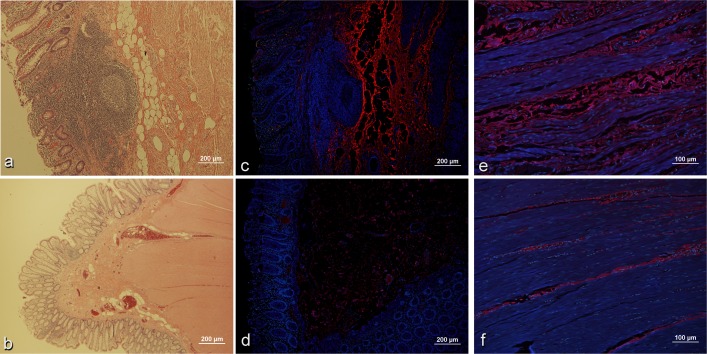
COMP presence in fibrostenotic Crohn’s disease and unaffected intestine. H&E staining of fibrostenoic (panel a) demonstrating smooth muscle hypertrophy and tissue architectural changes typical of Crohn’s disease, compared to normal unaffected intestine (panel b). Immunohistochemical staining for COMP (red), with DAPI nuclear background staining (blue), reveals accumulation within the submucosa and distorted muscularis (panels c, e) compared to minimal COMP staining tissue in normal intestine (panels d,f).

Immunofluorescence stains were performed to investigate whether COMP and HGFA were present within regions of fibrostenotic damage in resected intestine. Because full thickness tissue samples were not available from phenotypically inflamed non-stricturing subjects, fibrostenotic tissue sections were compared to normal margins of tissue resected for alternative indications (e.g. malignancy, diverticula disease). Qualitative assessment of H&E stains in fibrostenotic intestinal tissue sections revealed patchy lamina propria plasmacytosis, crypt distortion, fibrosis, and smooth muscle hypertrophy, typical of fibrostenotic Crohn’s disease, in all specimens. The degree of fibrosis was severe in all fibrostenotic tissue sections. There was also increased interstitial fibrosis in the muscularis propria of the fibrostenotic tissues. HGFA immunostaining signal was very weak in diseased tissue with little difference from normal control. COMP immunostaining signal was qualitatively much stronger in fibrostenotic tissue specimens, with only weak and nonspecific background staining in normal controls (Fig 2C & 2D). In addition, COMP signal co-localized with the fibrotic areas within the submucosa and muscularis propria of fibrostenotic tissue sections (Fig 2E and 2F).

## Discussion

In an attempt to non-invasively differentiate predominantly fibrostenotic from inflammatory intestinal injury, this study has highlighted COMP and HGFA among several glycoproteins identified by LC-MS/MS as potential serum biomarkers of fibrosis in Crohn’s disease. COMP has been well described in skeletal and connective tissue disorders, including pseudoachrondroplasia and osteochrondroplasias. While not previously associated with CD or intestinal damage, known aspects of COMP function highlight a role in fibrogenesis. COMP (also known as thrombospondin-5) is a member of the thrombospondin family of glycoproteins participating in extracellular matrix (ECM) production and tissue remodeling in response to injury [17]. COMP interactions with collagen, fibronectin, matrillin, and proteases (including MMP-3, -12, and -13) directly contribute to ECM development [18,19]. Transforming growth factor-β (TGF-β), a key regulator of myofibroblast activity and ECM characteristics, has been shown to induce COMP expression in human tissues [20]. Beyond direct contributions to the structural properties of ECM, COMP influences mesenchymal cell function, phenotype, and proliferation in response to ECM lattice components, also regulating collagen secretion into the ECM [21,22].

Despite its principal associations with connective tissue diseases, mounting evidence suggests a clinically relevant role for COMP in fibrogenesis within multiple organ systems. Cartilage damage associated with rheumatoid arthritis and osteoarthritis have been associated with changes in COMP expression [23]. COMP is overexpressed in dermal and pulmonary lesions in patients with scleroderma [20]. In idiopathic pulmonary fibrosis, Kaminski and colleagues reported a 13-fold increase of COMP expression in damaged lung tissue, also finding COMP serum levels were associated with pulmonary function decline as measured by forced vital capacity [24]. Elevations in serum levels of COMP have recently been shown to correlate with the presence and the degree of hepatic fibrosis by liver elastography (*r =* 0.71, *p*<0.001) [25,26]. COMP appears not only to have a mechanistic role in human fibrotic human diseases, but additionally is sufficiently secreted into blood to allow detection by conventional clinical methods.

Our results provide evidence that COMP is present in diseased fibrostenotic intestinal tissue in CD. Overlapping COMP and collagen staining within the diseased submucosa and muscularis of small and large intestine in the fibrostenotic cohort agrees with the well described interactions between COMP and ECM components. Serum elevations of COMP in fibrostenotic subjects persisted even following the resection of all diseased intestine. The constitutively elevated COMP expression observed could reflect a propensity for a fibrogenic response to tissue injury and inflammation. While more study is required, these results suggest that serum COMP levels could improve phenotype characterization, potentially serving as a predictive marker of future fibrostenotic complications. COMP could represent a marker of the rate of fibrogenesis, like a tachometer for fibrosis, rather than a marker of accumulated fibrotic bowel damage.

Hepatocyte growth factor activator (HGFA) was also elevated in fibrostenotic compared to inflammatory phenotype subjects. HGFA is a serine protease produced by the liver and secreted into blood as a zymogen (pro-HGFA) [27]. Its principal role is to activate hepatocyte growth factor (HGF) in response to tissue injury, along with matriptase and hepsin [28]. Interestingly, HGF has been well described as having anti-fibrotic properties. HGF is a growth and repair factor that is produced by several cell types, including fibroblasts, which inhibits epithelial to mesenchymal cell transformation and induces myofibroblast apoptosis [29]. HGF has a antagonistic relationship with TGF-β, favoring tissue regeneration and inhibiting fibrosis [30,31]. HGF has been shown to have anti-fibrotic effects in animal models of renal, cardiac, lung, and liver injury [32,33]. In fact, HGF is being explored as an anti-fibrotic therapeutic agent in pulmonary and cardiovascular disease [34]. Hepatocyte growth factor activator inhibitor-1 (HAI-1), which also modulates matriptase and matrix metalloproteases, is unsurprisingly associated with pulmonary and hepatic fibrosis [35,36]. Of note, HAI-1 expression in intestine is associated with increased intestinal permeability and susceptibility to inflammation in the DSS-colitis murine model [37]. HGFA elevation in the setting of fibrostenotic disease may reflect efforts to regenerate damaged intestine which are overcome by superimposed and dominating mechanisms of fibrogenesis within severely damaged intestine. The role of HGFA in CD remains unclear and further study is needed to understand its function and relevance as a clinical biomarker in IBD. Elevated HGFA levels in fibrostenotic subjects, with resolution following surgical resection, suggests it may serve as a surrogate biomarker of accumulated fibrotic bowel damage, much like an odometer for intestinal fibrosis.

Several limitations of this study must be acknowledged. A key limitation, hindering the majority of human intestinal fibrosis studies, is the absence of an existing gold standard measurement of fibrosis. Best efforts to define predominantly fibrostenotic vs. inflammatory CD phenotypes relied on objective CD activity parameters. However, despite CTE and MRE capabilities for characterizing disease anatomy and inflammatory activity, neither are reliable means for distinguishing inflammation from fibrosis. Several groups have shown that a radiologist global impression of stricture “activity” or “inactivity” was not associated with the presence or degree of fibrosis. In fact, increasing severity of inflammation has been associated with higher histologic grades of fibrosis [8]. The inflammatory group enrolled was unlikely to be entirely devoid of intestinal fibrosis and the fibrostenotic group exhibited appreciable inflammation, reflected by the trend of elevated CRP values. While convenient, CRP can identify the presence of inflammation, but does not perform well in defining the severity or burden of inflammation [38]. Further, CRP production in response to intestinal inflammation varies between individuals, owing to several factors including differences in promotor polymorphisms [39]. Considering these points in conjunction with the longer disease duration of the fibrostenotic subjects, we must concede that candidate biomarkers of fibrosis may also be correlated with overall disease burden and severity. However, the known biologic functions of COMP and HGFA corroborate their potential as markers to identify fibrogenesis and accumulated fibrosis in CD.

A second limitation is the heterogeneity of intestinal fibrosis composition in CD. While the length of resected intestine was not correlated with candidate biomarker expression, tissue pathology varies over the length of the diseased segment. Disease within surgically resected specimens may contain heavy ECM deposition surrounding deep ulceration or alternatively relatively less ECM deposition with more pronounced smooth muscle and neuronal proliferation. Variation of interstitial ECM deposition and smooth muscle hypertrophy within intestinal strictures may be an important covariate impacting protein expression profiles. Future studies are needed better describe this variation and its impact on post-operative course. A third limitation is the dynamic range detected by LC-MS/MS. Physiologically relevant changes in protein mass abundance may be difficult to detect in low-abundance proteins using the LC-MS/MS methods described. Our primary goal was to identify biomarkers detectable by common clinical laboratory assays. We therefore restricted analysis to glycoproteins with relatively high changes in mass abundance. While increasing the probability that candidate biomarkers would have a sufficient dynamic range to discern phenotypes by widely available ELISA, clinically relevant low-abundance proteins reflecting and quantifying fibrogenesis could have been missed in this preliminary glycoprotein survey. In addition, the variation observed between LC-MS/MS results and ELISA suggest that specialized detection methods may need to be developed for clinical use of these candidate biomarkers.

Despite the study limitations, COMP and HGFA present potential as biomarkers of fibrogenesis and fibrosis in CD. Blood-based quantitative biomarkers of fibrosis would provide an important complement to existing diagnostic and prognostic tools in IBD, and may prove to aid in longitudinal monitoring of both inflammation and fibrosis. Appreciation of the impact of fibrosis on CD course and management has fostered investigation of multiple imaging-based measurement methods [40]. Quantitative analysis of MRE T2-weighted signal and contrast enhancement washout, intestinal elastography, and collagen detection by magnetization transfer-MRI and photoacoustic imaging are being applied to address the need for fibrosis measurement tools [41–43]. Together with these emerging methods, COMP and HGFA could aid in non-invasively determining the histologic composition of intestinal disease. Knowing the degree of ongoing fibrogenesis or accumulated intestinal fibrosis may guide decisions between therapeutic intensification vs. proceeding to surgery in obstructive CD. In addition, as more therapeutic mechanisms become available, including agents interacting with IL-12/23, Janus kinases, and SMAD7 pathways, a more granular understanding of patient phenotype may better guide therapeutic choice and sequence. Finally, as anti-fibrotic medical therapies become available, measures of intestinal fibrosis will be needed for efficacy assessment. In conclusion, we present COMP and HGFA as serum-based biomarkers of intestinal fibrosis in CD. Future work is needed to replicate and further validate these findings and determine their ability to meaningfully monitor disease progression and predict future development of fibrostenotic disease complications.

## Supporting Information

S1 Appendix Expanded Proteomic Methods. (DOCX)

## Abbreviations

AUROC: area under the receiver operating characteristic
CBC: complete blood count
CD: Crohn’s disease
CHLE: cholinesterase
COMP: Cartilage oligomeric matrix protein
CRP: C-reactive protein
CTE: Computed tomography enterography
ECM: extracellular matrix
ELISA: enzyme-linked immunosorbent assay
FD: false discovery rate
FFPE: Formalin-fixed paraffin embedded
HBI: Harvey Bradshaw Index
HGFA: Hepatocyte growth factor alpha
MRE: Magnetic resonance enterography
POCE: procollagen C-endopeptidase
TGF-β: transforming growth factor-β
TMT: tandem mass tags
TNXB: tenascin-X
