# Supplementary material for: Serum Glycoproteome Profiles for Distinguishing Intestinal Fibrosis from Inflammation in Crohn's Disease
Source: PLoS One. 2017 Jan 23;12(1):e0170506. doi: 10.1371/journal.pone.0170506 (PMC5256928; doi:10.1371/journal.pone.0170506)
Supplement: S1 Appendix — (DOCX) [file pone.0170506.s001.docx]

**S1 Appendix. Expanded Proteomic Methods.**

**Detailed Proteomic Methods**

*Depletion of high-abundance serum proteins*

IgY-14 LC5 columns (Sigma, St. Louis, MO) were used to deplete 14 high-abundance proteins from patient sera. The depletion was performed with 100 µL serum according to manufacturer's instructions. The flow-through fraction was collected and transferred to IgY-14 LC10 columns (Millipore, Billerica, MA) and centrifuged at 4000g, followed by buffer exchange three times with 5 mL triethylammonium bicarbonate (TEAB). The protein concentration was measured using a Bradford assay kit (Bio-Rad, Hercules, CA).

*TMT labeling*

Depleted serum protein samples and internal standards were labeled with TMT reagents as described previously with some modifications. One hundred micrograms of proteins were reduced by 5mM TECP at 37 °C for 1 hr, and alkylated with 25 mM iodoacetamide for 30 min in the dark. The buffer was exchanged to 50 mM TEAB in 4 M urea with a final volume of 100 μL. TMT labeling reagent dissolved in 30 μL DMSO was transferred to protein samples, reacted for 2 h at room temperature, and quenched with 0.5% hydroxylamine for 15 min. Samples were combined and exchanged into lectin binding buffer for glycoprotein enrichment.

*Glycoprotein enrichment and enzymatic digestion*

Columns packed with 600 μL agarose-bound AAL were washed and equilibrated with 3 mL binding buffer (20 mM Tris, 0.15 M NaCl, pH = 7.5, protease inhibitor 1:100). TMT labeled samples in 1mL binding buffer were loaded onto columns and incubated for 15 min twice. The columns were washed with 5 volumes of binding buffer to remove unbound proteins. The captured glycoproteins were then eluted with four volumes of elution buffer (200 mM fucose in binding buffer). The elutes were concentrated and buffer exchanged into 50 mM NH4HCO3 using a 4 mL YM-3 filter. The extracted and TMT-labeled glycoproteins were then digested with trypsin (1:50) at 37 °C overnight. The N-glycans attached on asparagine (Asn) residues were released using PNGase F (New England Biolabs, Ipswich, MA) at 37 °C for 16 h. The samples were then dried with a SpeedVac concentrator (Thermo Savant, Milford, MA) and desalted using C18 spin columns (Thermo Fisher Scientific) prior to MS analysis.

*LC-MS/MS analysis*

TMT-labeled peptide mixtures were dissolved in 0.1% formic acid (FA) and loaded onto an Easy 1000 nano UHPLC system (Thermo) equipped with an Acclaim PepMap100 C18 Nano-Trap Column (75 μm x 2 cm) and an Acclaim PepMap RSLC column (75 μm × 25cm) coupled to an Orbitrap Fusion Tribrid Mass Spectrometer (Thermo Fisher Scientific). Peptides were separated with 0.1% FA in water (solvent A) and 0.1% FA in acetonitrile (solvent B) using an 85 minute linear gradient from 5 to 35% solvent B at a flow rate of 300 nL/min. The mass spectrometer was operated by taking one full MS scan followed by ten HCD MS/MS scans on the ten most intense ions from the MS spectrum. The resolution of full scans (m/z 350.0–1500.0) and HCD scans (fixed start from m/z 110.00) was set to 120,000 and 50,000, respectively. The AGC target value was set as 200,000 for the FTMS scan and 50,000 for the FTMS MSn scan.

Acquired MS/MS spectra were searched against the UniProt human database using SEQUEST in Proteome Discoverer 1.4 (Thermo Fisher Scientific). Searches were performed using the following settings: precursor ion m/z tolerance, ± 10 ppm; fragment ion m/z tolerance, ± 0.05 Da; two missed cleavages allowed; static modification, carbamidomethylation (+57.02146 Da, C) and TMT 6-plex (+219.163 Da) of lysines and protein N-termini; dynamic modifications: oxidation (+15.99492 Da, M) and deamidation (+0.98402 Da, N). Identified peptides were filtered using a 1% peptide-level false discovery rate (FDR) and quantification was performed using reporter ions.
